# Negotiating severity behind the scenes: prenatal testing in Germany

**DOI:** 10.1038/s41431-024-01612-z

**Published:** 2024-04-27

**Authors:** Tamar Nov-Klaiman, Hilary Bowman-Smart, Ruth Horn

**Affiliations:** 1 Institute of Ethics and History of Health in Society, University of Augsburg, Augsburg, Germany; 2 Australian Centre for Precision Health, Clinical and Health Sciences, University of South Australia, Adelaide, Australia; 3 Biomedical Ethics Research Group, Murdoch Children’s Research Institute, Melbourne, Australia; 4 Ethox Centre, Nuffield Department of Population Health, University of Oxford, Oxford, UK

**Keywords:** Scientific community and society, Ethics

## Abstract

Foetal-related severity is a key concept in policy and legislation relating to access to both reproductive technologies and selective abortions in many countries around the world, but not in Germany. This study sheds light on how ‘severity’ in the context of prenatal testing is understood and negotiated within the particular socio-cultural and legal context of Germany, where ‘severity’ relating to foetal clinical findings neither counts as a justification to implement population prenatal screening programs, nor as a legal ground to terminate pregnancy. This study explores the views of women who undergo prenatal testing, as well as of professionals who encounter them, through semi-structured interviews. It showcases how they frame severity and questions whether the existing legal and regulatory framework relating to prenatal testing and termination of pregnancy addresses their concerns and needs regarding reproductive decision-making. The interviews (*n* = 27) reveal that despite it being legally outside the explicit reasons for testing and termination of pregnancy, both women and professionals negotiate severity behind the scenes. Their interpretation of severity is highly context-dependent and relies on clinical, social and familial facets. Their perceptions of severity guide them in their handling of and decision-making around pregnancy management. Acknowledging the personal nature of severity assessment and providing professional or legal guidance which explicitly mentions foetal anomaly as a legitimate factor in pregnancy management could provide healthcare professionals and patients with the room needed to manage the pregnancy favourably.

## Introduction

Severity of foetal anomaly is a key concept in reproductive technologies, be they preconception carrier screening, pre-implantation diagnostics, or prenatal testing. Legal, ethical and policy documents relating to reproductive technologies and access to selective pregnancy termination frequently use ‘severity’ or ‘seriousness’ as a central criterion when setting the boundaries for the medical conditions for which these technologies should be applied [1]. However, no clear legal or social definition exists for severe or serious conditions, making the use of the term vague and subjective [2]. While some studies attempt to provide taxonomies for the evaluation of disease severity based on clinical characteristics [3], others show that the perception of severity is largely shaped by economic, social and cultural factors, as well as personal experience [1, 4–6].

It is therefore critical to examine the ways that severity is understood in a range of cultural contexts. Intending to shed more light on how severity is understood and conceptualised, the present work examines severity in the context of prenatal testing within the particular German socio-cultural, historical and legal setting. Germany has been described as possessing a unique ethical, legal and policy landscape in relation to reproductive technologies and pregnancy management [7, 8]. In contrast to countries such as France and England, where ‘severe’ foetal anomalies provide a legal ground for termination of pregnancy (TOP) without gestational limit [9, 10], in Germany, foetal anomaly itself is not grounds for accessing TOP. A reform of the German abortion law in 1995 abolished the ‘embryopathic indication’, i.e. the criterion which permitted TOP on the explicit grounds of foetal anomaly [11]. Like much of German policy and legislation, this was in part driven by a desire to differentiate Germany from historical eugenic practices [12].

Currently, TOP is permitted before 12 weeks gestation (following pregnancy counselling), and for ‘social-medical’ reasons at any gestation, 'to avert a danger to the life or danger of a serious impairment of the physical or mental state of health of the pregnant women' (German criminal code section 218a [2]). Although embryopathy can no longer be directly grounds for TOP, it can nonetheless still be framed as a medical indication on the basis that it threatens the woman’s wellbeing. In other words, severity of a foetal anomaly is to be assessed by the impact on the woman. However, while data on TOP in Germany are relatively scarce, evidence suggests that decisions around TOP at later gestations are still largely driven by the presence of foetal anomaly [13].

Similarly, in Germany, foetal anomalies do not justify prenatal screening programmes targeting a specific genetic condition, such as the combined-first trimester screening (CFTS) or non-invasive prenatal testing (NIPT) that can identify an increased chance of the common trisomies T21, T13 and T18. Unlike other countries, such as England and France, that offer NIPT within the public services to all pregnant women with a higher chance of a trisomy based on CFTS, Germany opted to offer NIPT on a case-by-case approach, when the possibility of a trisomy presents an 'unreasonable burden' for the woman [8, 9, 14]. Basing the offer on an assessment of the woman’s well-being echoes the German decision to allow TOP on that ground. Here too, the severity of the situation is measured not by medical criteria and the impact of the condition on the future child, but by the–current and expected—impact on the woman.

At the backdrop of this regulatory framework, it is important to explore the views of those who operate within it. Through interviews with German professionals who depict their own views and portray those of their patients, alongside interviews with women, we explore perceptions of severity in the context of prenatal testing. This is particularly interesting within the German context where neither TOP nor prenatal screening programmes explicitly on the grounds of foetal-related severity are legal, yet severity is negotiated behind the scenes and its understanding affects professionals’ and women’s attitudes and decisions, whether choosing TOP or preparing for the birth of a child with special needs.

## Methods

This paper is part of a wider comparative empirical bioethics project exploring the ethical issues arising from the introduction of NIPT into routine care in England, France and Germany.

The present paper is based on interviews conducted in Germany. It analyses how the concept of severity is understood and used by professionals and women within the German regulatory framework.

### Data collection

In Germany, professionals were recruited through pre-existing networks within prenatal genetics and policy, followed by subsequent snowball sampling. Women were recruited by posting the invitation on the websites of patients’ organisations providing information about NIPT, such as the Down Syndrome Association and through flyers put out in the clinics of some of the professional participants.

27 semi-structured interviews were conducted in German online via Microsoft Teams by two qualitative researchers, RH and HBS, between June 2021 and February 2022.

The professionals’ (*n* = 20) expertise includes obstetrics and gynaecology; foetal medicine; pregnancy or prenatal counselling; clinical genetics; and policy. The focus of the broader study is on NIPT: Women participants (*n* = 7) were 30–50 years old and users of prenatal tests (six of them used NIPT in at least one of their pregnancies). Five women were pregnant at the time of the interview. Former pregnancies of three of the participants were terminated following abnormal test results. While recruitment of professionals was pursued until saturation was reached and no new themes emerged, we continued recruitment of women until our internal project deadline.

Using separate interview guides for professionals and women, interviewees were probed to elaborate on their perceptions and concerns—specific to their role as either professional or patient—relating to NIPT and pregnancy management (see supplementary files).

### Data analysis

The interviews were transcribed verbatim while removing participant’s identifiers. They were then coded and analysed using NVivo software. To facilitate reading, coding and discussing of themes by the authors, the translation of the interview transcripts into English was completed by RH, who is a native German speaker, with the assistance of translation software. RH re-reviewed and validated the accuracy of the translation for the purpose of coding as well as for the use of selected quotes presented in the 'Results' section. Following a thematic analysis approach [15], emerging broad themes and subsequent subthemes were identified. In periodical meetings, TNK and RH discussed the relevance of the codes identified and agreed on needed modifications and reclassifications. They discussed new findings when they appeared and their connection to the identified codes, thereby preventing the potential bias of a single rater.

## Results

'I would define a serious illness as one with a lasting impact on the individual, i.e., an illness that cannot be cured and may have a lasting impact on the quality of life'.

[Prof_18]

With this clarity, one foetal specialist described their interpretation of ‘severity’. However, associating severity with quality of life leaves room for interpretation.

Interviewees discussed severity or seriousness not solely by explicit use of the terms, but often as an implicit underlying factor guiding decision-making around testing and TOP. Three major elements emerged in the way that German professionals and women understand and assess the severity of a foetal anomaly. The first element is the clinical aspect (e.g. clinical characteristics and life expectancy); the second is the impact on the family (emotional and practical); and the third is the social aspect (e.g. the attitudes of the environment and the ability to participate in social life).

### The clinical perspective

The clinical pillar of severity in the prenatal setting relies on the detection of abnormal findings. However, even a definite diagnosis does not always imply a clear prognosis. Due to attributes such as incomplete penetrance and variable expression, the prenatal detection of many conditions cannot sufficiently inform about the expected clinical manifestation and its severity.

Some interviewees referred to the inability to predict the clinical picture as a driving factor when opting for TOP. Without prognosis, prospective parents feel frustrated and are left with only the imagination to assume severity and what life would look like. In the words of a pregnant woman whose first son was born with a genetic condition, and her second pregnancy was terminated after detecting Down syndrome:

'I discussed that with our geneticist […]. I said: ‘Can’t you tell me what will become of this child? And who am I to decide whether or not this child has a life worth living?’ Because that is what matters to us most.' [PAT-013]

One specialist in obstetrics and gynaecology demonstrated the importance imagination plays when no precise prognosis can be obtained, and described patients’ reaction to that:

'There is a large spectrum [of clinical expression] and also studies are showing that women and couples always assume the worst in their case […]. So, there’s a lot of fear of being exposed to a life change that you don’t want and can’t handle.'

[PROF-010]

In some instances, ‘suffering’ following certain conditions was the describing term—a measure of severity used as a justification for testing and TOP. However, as one patient representative, member of a policy group and mother of a disabled child herself put it–the point beyond which suffering is inflicted is hard to define:

'[Some conditions] are associated with a lot of suffering for everyone, and I think it’s just not possible to mark boundaries. […] I still have this ‘illness and disability as suffering’, and ‘suffering to be avoided’ in my head […] That’s how I was raised.'

[PROF-012]

These excerpts demonstrate that severity of foetal anomaly, despite it being ignored by law, plays an important role in how women and professionals think about continuation of an affected pregnancy.

In practice, the current framework dictates that much room is given to assessment by the treating physician, potentially at the expense of women’s preferences, as one professional put it:

'A medical indication must be issued by a doctor. Ultimately it is not the couple who decides about it [TOP]. At best that’s consensual. Some couples think that if something is found, they can decide. That’s not true. Usually there is a conversation, and you and the doctor then come to a common stance on how to proceed, but the couple doesn’t decide. Sometime there is controversy.'

[PROF-016]

### The familial context

When talking about the scenario of a future child with a medical condition, the interviewees often referred to the impacts on all family members–emotional and practical–as fundamental to assessing severity.

Family resilience in the face of having a child or a sibling with a medical condition was a recurring theme when negotiating severity, as mentioned by both professionals and women. This included questioning the parents’ ability to withstand the pressures resulting from caring for a sick child and maintaining other aspects of their life and identity. Another concern was the impact on the siblings who are 'pushed aside' when much of the energy and time are directed at the sick child and who might later carry the responsibility when the parents are no longer there. All these concerns translated into decisions around testing and TOP. One obstetrician described a spectrum of arguments patients give when they opt for a pregnancy termination:

“With Down syndrome, it’s more like, ‘I don’t want to do this to the kid if I’m dead. Who will take care of the child?’ ‘I don’t want to do that to the siblings who would then have to be the caregivers themselves [and] who are pushed aside because I only care about this child.’ ‘I might see the partnership in danger because it’s an insane burden.’ ‘I’m overwhelmed because I’m a single parent. Don’t have any backing or anything.’” [PROF-026]

One of the interviewees, a pregnant woman, explained why she decided to take up NIPT:

'We already have one child. If our second child were to have a disability, then of course that would have an even greater impact on family life. So, it was very important to us that we can plan sensibly. We wanted to know, because it would also affect our first child and my options for going back to work. In other words, our entire everyday life.' [PAT-025]

Other concerns, of a more practical nature, included financial aspects and the fear of lacking means to support a child with special needs. One woman explained:

'I believe that every person has a right to live, no matter how they are. But I also believe that as a parent you can certainly decide: ‘Am I able to? Do I dare? Do I have the time and money?’ It is unfortunately also a question of money – The money to get my child through life despite the handicap.'

[PAT-023]

### The social context

Beyond the clinical expression and the impact on family members, the social sphere, namely participation in social life, was another key element in assessing ’severity'. The expected social exclusion of a child with disability and family members was a frequent concern raised by both professionals and women. This scenario is integrated within the considerations underlying pregnancy management. A former gynaecologist, currently working as a counsellor for prenatal diagnosis, emphasised what patients voice:

'It is actually the case that life with a child with a disability also means to a certain extent that you are excluded. This is a concern most parents have.'

[PROF-016]

Interviewees also discussed the impact of cultural norms when deciding what it means for a foetal anomaly (or trait) to be severe enough to warrant TOP. One obstetrician reported:

'There are people who, when they have a girl after three girls, feel that the fourth girl is already difficult because she is not the boy they longed for. We shake our heads [yet, … this scenario] is a catastrophe in families from certain cultures.' [PROF-027]

Our interviews confirm how severity is measured against the development and implementation of prenatal technologies. Interviewees described how the cultural narrative around a diagnosis is strongly shaped by the degree to which it is considered ‘foreseeable’ and ‘avoidable’. The use of prenatal testing is the result of social standards but at the same time shapes them, changing the cultural standard for what is considered severe and to be avoided. One prenatal diagnostician described how technology and social expectations are intertwined:

'Women [parenting a child with Down syndrome] are already telling they hear sayings like: ‘Well, that shouldn’t have happened. There are options there [to avoid it].’ So surely the introduction [of NIPT] as a health insurance benefit will make a condition like Down syndrome even more unworthy of life than before.'

[PROF-010]

Another vivid account of the role culture plays in the relations between technology and social standards was given by a foetal specialist:

“I always say: Imagine we live on an island and someone with Down syndrome is worshipped there as a deity. Then people would be happy if they had such a child. But the opposite is the case. Everyone recognises a child with Down syndrome and as a parent you are always in this position of justification and must fear that you will be asked: ‘Well, how did that happen? Couldn’t you foresee that?’” [PROF-021]

Some respondents feared that social standards of severity and the development of improved prenatal tests could infringe the ‘right not to know’ and put pressure on parents to test for anomalies.

Yet, at the same time, the interviews described an opposite pressure that impacts severity assessment and pregnancy management; the taboo of terminating a pregnancy based on the diagnosis of foetal anomaly. This, together with a limited number of professionals offering TOP, makes it difficult for women to access TOP on these grounds:

“I wish that abortion was not a taboo. We live in an open society; it is legal. So why make such a taboo out of it? 'It is an individual’s decision to make this ethical choice of what suits them, what is justifiable.'”

[PAT_20]

'The supply of abortions from week 14+ is really bad in Germany. So, women go abroad.' [PROF-011]

## Discussion

This paper sheds light on how ‘severity’ in the context of prenatal testing is understood and negotiated within the particular context of Germany. This is of special interest, since foetal-related severity is a key concept in policy and legislation relating to access to both reproductive technologies and selective abortions in many countries around the world, but not in Germany.

### Contextuality of severity

Our findings show that severity is often an underlying guiding factor in decision-making around testing and TOP, for both professionals and women, even when the term is not explicitly used in legislation. The analysis revealed similarities in the way that participants from both groups understand and assess the severity of a foetal anomaly. Our participants framed severity from a medical, social and familial perspective while emphasising the uncertainty and distress around the inability to predict prenatally what the real impact would be; thus, relying on imagination for decision-making around the future child’s existence. This arguably makes the case of severity assessment in the prenatal setting unique.

The study shows that perception of severity is very personal, subjective, and connected to what women feel capable of dealing with in their particular situation. Dive et al (2023) describe the importance of distinguishing between a more generalised understanding of the ’severity' of a condition, and the ’severity' of a condition in a particular instance [16]. Our results underscore the importance of this in the prenatal setting. When clinicians draw on the concept of ’severity', it is important that they integrate the patients’ input to respect their life circumstances, preferences and values. This is echoed by a number of studies emphasising the subjectivity of how severity is understood and the difficulty to capture it by apparently objective criteria [1, 4, 5].

Our interviewees contemplated severity based on a combination of factors—clinical, familial and social—shaping the lived experience and thereby the understanding of severity. Balanced information on life with disability, including the fact that severity depends on each person’s views and experiences, should be communicated to women in prenatal counselling, since informed decision-making mandates 'comprehensive information about the potential rewards and challenges of living with or raising a child with, a disability' [17].

Given the different weight individuals assign to each factor in their personal life circumstances, it is clear why no ‘one-size-fits-all’ kind of definition could cater to the varying nature of severity perception. The legal and regulatory framework relating to prenatal testing and termination of pregnancy must be, therefore, flexible enough to safeguard women’s autonomy. We ask whether the unique German framework supports this goal.

### The embryopathic indication, prenatal screening programmes, and ’severity'

The embryopathic indication that was in place in the German abortion law until 1995, allowed women to terminate pregnancies on the grounds of foetal anomalies. With its abolishment, the focus for TOPs after 12 weeks of conception shifted from a foetal indication to an indication that was only related to the woman. In other words, the formal object of severity assessment was replaced. To comply with the German criminal code section 218a [2], a medical opinion must be given, to prove that there is 'a danger to the life of or a danger of grave impairment to the pregnant woman’s physical or mental health' if the pregnancy continues. The condition of the foetus, however severe it might be, does not in itself justify access to TOP.

The case is different, however, for extra-corporeal embryos in the context of preimplantation genetic diagnosis (PGD), where embryo selection on the basis of severe conditions is legal [18, 19], thereby possibly allowing the use of PGD to select, among others, against aneuploidies [20]. The existence of two distinct frameworks—one for the extra-corporeal embryo and one for the intra-corporeal foetus—is intriguing, yet perhaps reflective of the German society. PGD is dependent upon IVF procedures and cannot, therefore, serve as a population screening method. Moreover, TOP following the detection of abnormal findings could be more frowned upon since it occurs at a much more advanced foetal development compared to the embryonic stage in PGD, connecting it to the broader debate about the ethics and social acceptability of TOP in German public discourse. These characteristics possibly make TOP less tolerable and could be the impetus underlying the German legislation, reflecting a society that desires to differentiate itself from historical eugenic practices.

The abolishment of the embryopathic indication from the abortion law led to uncertainty among physicians and lawyers [21] and potential conflicts with law enforcement authorities [13], possibly resulting in physicians hesitating to assist women in terminating in the case of foetal anomaly. Furthermore, the taboo around foetal anomaly and the lack of legal recognition of it being an influential factor in the decision-making process arguably discourages professionals to provide terminations beyond 12 weeks of gestation. Indeed, according to our interviews and other studies, accessing TOP in Germany—especially later terminations, which are strongly connected to abnormal findings—is difficult, forcing some women to drive to neighbouring countries to get the service [22]. Our interviews and other studies [22, 23] indicate, however, that a major fraction of terminations after 12 weeks gestation involve foetal anomaly.

This could be one possible explanation for the increase in first-trimester abortions that has been observed in Germany and recently reported [24, 25]. It aligns with the increase observed in NIPT uptake [26] and could potentially point to women opting for early TOP based on NIPT without confirmatory diagnostic testing which is performed beyond the 12 weeks gestation limit [27]. If this is indeed the underlying reason, this trend may pose concerns. NIPT, which investigates placenta-derived DNA, is fairly accurate in detecting common aneuploidies, especially trisomy 21, but has high false positive rates for other conditions, such as sex chromosome aneuploidies [28]. Therefore, women who opt for TOP following abnormal NIPT results without verifying them in diagnostic testing—in order to save time or to avoid the difficulties of accessing TOP later in pregnancy—might make pregnancy decisions based on inconclusive evidence.

Failing to acknowledge foetal anomaly as a legitimate factor in decision-making around TOP perpetuates the stigma and shame around selective TOP and leaves much room for professionals to assess according to their own views whether the threshold of 'grave impairment' to the woman is met. This may conflict with the views of patients [29]. Clinicians may focus on biomedical aspects of the condition and be driven by their own values, rather than by the patient’s perspectives and context. Indeed, some of our respondents articulated how in the current situation, women’s perceptions and preferences could be overruled by professionals, potentially making access to TOP more difficult, thereby undermining women’s reproductive decisions and therefore autonomy. The same goes for accessing NIPT according to the current policy, where no clear cut-offs define it, but rather a subjective feeling that requires the approval of a doctor [8].

In order to allow women to be the decision-makers according to their situation and to legitimise the decision to terminate on the grounds of foetal anomaly, clear professional or legal guidance should explicitly mention that a diagnosis of foetal anomaly can impact women’s health and wellbeing. This would align with the guidelines regulating the access to NIPT in Germany, which state that access is justified when 'the possibility of a trisomy burdens a woman so much that she wants it clarified' [30].

While one could argue that explicit reference to foetal anomaly in guidance could increase the social pressure on women to avoid disability, we see that in practice, even without such reference there is no escape from negotiating severity and considering social responses during the decision-making process. The current law seems to come short in both senses: it does not shield women who choose to keep pregnancies with a diagnosed foetal anomaly from the associated social pressures, and at the same time it does not support women who wish to terminate affected pregnancies.

When too much room is given to assessment by the treating physicians—as is currently the case—there is also fear that women will not receive a uniform treatment throughout the country. Stronger guidance and recognition of foetal anomaly as a criterion to access TOP along with the implementation of population screening programmes as a standard of care for those who want it could help level the situation across Germany. Testing and subsequent pregnancy decisions would be more straightforward and less subject to physicians’ inclinations.

A primary intention behind the abolishment of the embryopathic indication and the offer of NIPT on a case-by-case basis was to fight against value judgements about life with disability [21]. Yet, our interviews as well as other studies [31, 32] show that women do not base pregnancy decisions on judgments about the worthiness of lives of people with disability, but rather focus on what they feel able to deal with personally and what life they would like to offer to their offspring. Indeed, the Nairobi principles affirmed that 'individual choices about one’s own pregnancy are not eugenics, and nobody exercises discrimination when making choices about their own pregnancies' [33]. It is through this lens that severity should be negotiated.

Our findings echo the literature that recognises that the ’severity' or ’seriousness' of any foetal anomaly in prenatal care cannot be understood in isolation, but rather requires a context-dependent understanding. They also demonstrate that removing references to foetal anomaly in legislation, with it remaining the focus in actual practice, is flawed.

## Study limitations

The study is based on a small group of respondents, especially that of the women interviewed, thus not allowing for generalisation. This limitation is, however, characteristic of qualitative studies. They allow, instead, for in-depth exploration and insights. The small group may have led to missing a broader range of viewpoints and therefore serves as an exploratory study. Future studies should include larger samples of women, as well as professionals.

## Conclusion

The German case shows that removing criteria such as severity from legislation or policy misses its symbolic value and raises obstacles in practice. Our findings show that without explicit reference to foetal-related severity, patients and professionals are nonetheless guided by it as a key criterion when deciding regarding testing and TOP. However, with its absence they are not able to openly discuss what a foetal anomaly would mean for their life, struggling to make decisions that suit their life circumstances and values, are not shielded from social pressures, and are exposed to inequities in care. Acknowledging the importance foetal anomaly plays in the decision-making while providing strong guidance on the importance of interpreting ’severity' as highly context-dependent could provide healthcare professionals and patients with the room needed to manage the pregnancy favourably.

## Supplementary information

Interview guide - Women

Interview guide - Professionals

## Data Availability

Data are available from the UK Data Archive for researchers who meet the criteria for access to confidential data: Horn, Ruth (2023). Non-invasive Prenatal Testing Study: Comparison England, France, Germany, 2021–2022. [Data Collection]. Colchester, Essex: UK Data Service. 10.5255/UKDA-SN-856508. https://reshare.ukdataservice.ac.uk/856508/.
